# Knochenstimulation 4.0 – Kombination aus EMTT und ESWT bei Humeruspseudarthrose: Ein Fallbericht

**DOI:** 10.1007/s00113-021-01025-3

**Published:** 2021-06-16

**Authors:** Karsten Knobloch

**Affiliations:** SportPraxis Prof. Knobloch, Heiligerstr. 3, 30159 Hannover, Deutschland

## Anamnese

Eine 36-jährige angeschnallte Pkw-Beifahrerin auf dem rechten Rücksitz verunfallte im August 2019 (Differenzgeschwindigkeit Delta v 70 km/h) mit Spiralfraktur des Humerus rechts in der 19. Schwangerschaftswoche. Seinerzeit konservative Therapie mit gipsverstärktem Gilchrist.

## Befund

Bei fortdauernden Schmerzen und Funktionseinschränkung im Alltäglichen am 18.02.2020 Oberarm-CT mit Nachweis einer Humeruspseudarthrose (6 Monate nach Unfall) mit um Schaftbreite versetztem Verlauf.

## Diagnose

**Figure 1:**
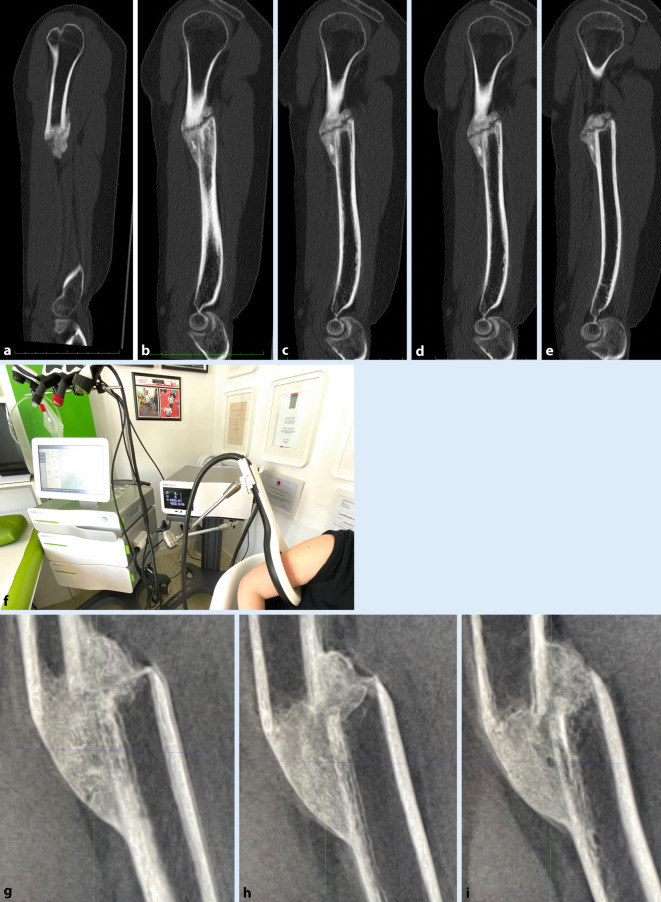

Humeruspseudarthrose (Abb. 1).

## Therapie

Am 18.03.2020 Beginn der apparativem Knochenstimulationstherapie als neuartige Kombination als individueller Heilversuch im Einverständnis mit der Patientin aus:

1. hochenergetischer fokussierter extrakorporaler Stoßwellentherapie (ESWT, Storz Ultra mit einem elektromagnetischen Generator 0,3 mJ/mm^2^, 3000 Impulse) gemäß den evidenzbasierten Leitlinien u. a. der DIGEST (www.digest-ev.de),
2. extrakorporale Magnetotransduktionstherapie (EMTT, Storz Magnetolith, Energiestufe 8/8, 8 Hz, 6000 Impulse/Sitzung). Die EMTT ist eine Weiterentwicklung der bekannten „pulsed electromagnetic field therapy (PEMF)“, für die positive Effekte auf die Knochenheilung [1–3] wie auch für die Osteointegration von Implantaten [4, 5] beschrieben sind – beide benutzen oszillierende Magnetfelder. Jedoch unterscheiden sich EMTT-Geräte von den PEMF-Geräten in 2 wesentlichen physikalischen Parametern:
  1. Die Oszillationsfrequenz ist beim EMTT mit 7 µs vs. 280 µs beim PEMF ungleich schneller und hochfrequenter. Damit Magnetfelder eine biologische Wirkung erzielen, ist die Überwindung einer „Schwellendosis“ von 10 mT nötig: Ein einzelner Impuls eines EMTT-Gerätes erreicht dies 10-mal in 70 µs, ein PEMF-Gerät genau einmal in 280 µs.
  2. Die elektromagnetische Transduktionsleistung, gemessen in kT/s, ist bei PEMF-Geräten definitionsgemäß < 60kT/s, bei EMTT-Geräten > 60kT/s – sprich die Leistung der EMTT-Geräte ist ungleich höher im Vergleich zu PEMF-Geräten.

Diese Kombination aus fokussierter ESWT und EMTT wurde 3‑malig im Abstand von einer Woche im März/April 2020 während des ersten Coronalockdowns ambulant durchgeführt, mit 95,9 J Gesamtenergie. Am 06.05.2020 Humerus-Dünnschichtbilddiagnostik als digitale Volumentomographie (SCS MedSeries H22). Hier erfreulicherweise keine Frakturlinie der Pseudarthrose mehr nachweisbar, sprich Konsolidierung der Humeruspseudarthrose bereits 5 Wochen nach der letzten Knochenstimulationsbehandlung. Die Funktion des Armes konnte daraufhin im Alltag wiederhergestellt werden, mit einem DASH-Score von 12 Punkten im Juli 2020, acht Wochen nach der Bildgebung (von 63 Punkten kommend im März).

## Fallanalyse

Pseudarthrosen des Humerus treten in bis zu 15 % der Humerusfrakturen auf [6]. Jüngst wurden in dieser Zeitschrift die konservativen Therapieoptionen von Pseudarthrosen von Großner und Schmidmaier lesenswert beleuchtet [7]. Der Einsatz der fokussierten Stoßwellentherapie (ESWT) zur Behandlung von Pseudarthrosen der langen Röhrenknochen ist seit den frühen 1990er-Jahren u. a. durch die Habilitationsschrift von Prof. Ekkernkamp [8] in Bochum und weiteren intensiv studiert und beschrieben [9].

Lokalisationsabhängig wurden aber deutliche Unterschiede der Erfolgsraten berichtet. So erreichte man eine knöcherne Konsolidierung bei Humeruspseudarthrosen mit fokussierter hochenergetischer ESWT mit dem Ossatron von HMT (2500–4000 Impulse, 0,25–0,4mJ/mm^2^) anfangs bei nur einem von 5 behandelten Patienten [10] – sprich in 20 %. In einer Kohorte mit 13 Humeruspseudarthrosen [11] im Jahr 2009 wurde die hochenergetische fokussierte Stoßwellentherapie mit 4000 Impulsen bei etwas höherer Energieflussdichte von 0,56mJ/mm^2^ mit dem vorgenannten Ossatron von HMT (Schweiz) angewendet, mit einer Therapiesitzung in örtlicher Betäubung. Die Heilungsraten der Humeruspseudarthrosen waren zeitabhängig 2 Monate nach ESWT 8 % (1/13), 3 Monate nach ESWT bei 23 % (3/13) und 6 Monate nach ESWT bei 62 % (8/13), mithin offenbar besser als die eingangs berichteten 20 % Erfolg.

In dieser Zeitschrift berichtet die unfallchirurgische Klinik der Medizinischen Hochschule Hannover (MHH) im Jahr 1999 [12] von 27 Pseudarthrosen an langen Röhrenknochen (11 bereits mit Reosteosynthesen), mit einer Erfolgsrate von 41 % nach 6 Monaten durch die 2‑malige fokussierte ESWT (0,2mJ/mm^2^) an langen Röhrenknochen. Von den 2 eingeschlossenen Oberarmpseudarthrosen kam es nur in einem Fall (metaphysär, 24 Jahre alt, ESWT 28 Wochen nach Operation) zur Ausheilung, sprich 50 %iger Erfolgsrate am Humerus.

**Figure 2:**
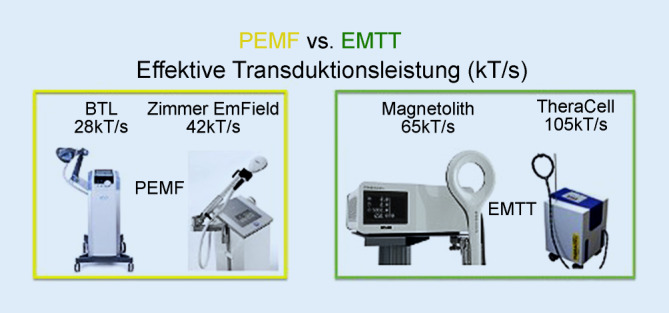

Die Ergänzung der EMTT über oszillierende Magnetfelder zeigt in diesem Fall eine rasche Konsolidierung der Humerusschaftpseudarthrose. Bei Pseudarthrosen des Kahnbeins und der Mittelhand ist der kombinierte Einsatz der fokussierten ESWT und EMTT jüngst beschrieben [13, 14]. Im Unterschied zur „pulsed magnetic field therapy (PEMF)“ mit einer effektiven Transduktionsleistung < 60mT/s zeichnen sich EMTT-Geräte wie das Storz Magnetolith® durch eine effektive Transduktionsleistung > 60kT/s und eine 40-fach höhere Oszillationsfrequenz im Vergleich zur PEMF aus – sprich, es bestehen physikalische Unterschiede in der biophysikalischen Gerätetechnik (Abb. 2).

Der weitere Einsatz der EMTT in Ergänzung oder auch allein zur Knochenstimulationstherapie ist in prospektiven Studien zu prüfen.

## Fazit für die Praxis

- Die Kombination der fokussierten ESWT und der extrakorporalen Magnetotransduktionstherapie (EMTT) scheint die Knochenheilung bei Humeruspseudarthrosen rasch zu stimulieren.
- Es handelt sich um eine nichtinvasive, ambulant und schmerzfrei durchführbare Therapie, auch bei einliegenden „Magnet(MR)-sicheren“ Implantaten, ohne Nebenwirkungen.
